# Mutations of *ABCD1* gene and phenotype of Vietnamese patients with X-linked adrenoleukodystrophy (X-ALD)

**DOI:** 10.1186/1687-9856-2013-S1-P127

**Published:** 2013-10-03

**Authors:** Vu Chi Dung, Nobuyuki Shimozawa, Nguyen Ngoc Khanh, Bui Phuong Thao, Can Thi Bich Ngoc, Nguyen Phu Dat, Nguyen Thi Hoan

**Affiliations:** 1 Vietnam National Hospital of Pediatrics, Hanoi, Vietnam; 2 Division of Genomics Research, Life Science Research Center, Gifu University, Department of Pediatrics, Gifu University School of Medicine, Gifu, Japan

## 

X-linked adrenoleukodystrophy (X-ALD) is caused by a defect in the gene ABCD1, which maps to Xq28 and codes for a peroxisomal membrane protein that is a member of the ATP-binding cassette transporter superfamily. X-ALD is panethnic and affects approximately 1:20,000 males. This disease characterized by progressive neurologic dysfunction, occasionally associated with adrenal insufficiency.

We aim to describe the clinical, laboratory and cerebral MRI characteristics of Vietnamese patients with X-ALD and to identify mutations of ABCD1 in these cases. Clinical features, biochemical finding and cerebral MRI lesions of 9 cases from 7 unrelated familes were studied. Genomic DNA from these patients was extracted using standard procedures from the peripheral blood leukocytes. Mutation analysis of ABCD1 was performed using Polymerase chain reaction (PCR) and DNA direct sequencing.

Among these patients, two families had two children with X-ALD, the others were unrelated but one case had family history of X-ALD (his maternal grand mother has a sister whose two sons having paralysis for more than 1 year, hyper-pigmentation and died at the age of 7 and 12 yrs). Endocrinology symptoms of adrenal insufficiency were observed in 8/9 cases; 7/9 cases showed neurological symptoms of cerebral ALD or adrenomyeloneuropathy; 2/9 cases had only symptoms of chronic adrenal insufficiency and no neurological symptoms until 12 and 5 years of age, respectively. 8/9 cases had serum cortisole and ACTH measured confirmed adrenal insufficiency. 8/8 cases showed increased plasma VLCFA. Neuroimaging studies (cerebral MRI) showed classical posterior pattern in 7 cases who had neurological symptoms and normal pattern in 2 cases without neurological manifestations. We identified 7 different mutations of ABCD1 in 9 patients. Of which, four novel mutations [c.1202G>T (p.Arg401Trp); c.1208T>A (p.Met403Lys); IVS8+28-551bp del; and the extent of deletion included between IVS1+505 and IVS2+1501, containing whole the exon 2 (4243bp), plus insertion of 79bp from BAP31 and 8bp from unknown origin in this deleted region] were identified in four unrelated patients with neurological symptoms. The reported mutation c.1628C>T (p.Pro543Leu) was identified in two cases (sibling: elder had no neurological symptoms and younger had progressive neurological disability). The reported mutation c.1553G>A (p.Arg518Gln) was found in a boy without neurogical symptoms at 5 years of age. The reported mutation c.1552 C>T (p.Arg518Trp) was identified in two cases (sibling: both have adrenal insufficiency and neurogical symptoms).

For the first time, mutations in ABCD1 are identified in X-ALD Vietnamese patients. Despite many mutations having been identified in patients with these clinical phenotypes, the genotype-phenotype correlations have not been clarified.

